# Diphtheria, Its Nature and Treatment

**Published:** 1861-03

**Authors:** 


Diphtheria; Its Nature and Treatment, with an account of the History of its prevalence in various countries. By Daniel Denison Slade, M. D., being the Dissertation to which the Fiske Fund Prize was awarded in July, 1860. Philadelphia: Blanchard & Lea, 1861.

This is one of the best Monographs that has yet appeared on the subject of Diphtheria.

For sale at the excellent Book Establishment of W. B. Keen & Co., Lake street, Chicago.

